# Upregulation of Human Endogenous Retroviruses in Bronchoalveolar Lavage Fluid of COVID-19 Patients

**DOI:** 10.1128/Spectrum.01260-21

**Published:** 2021-10-06

**Authors:** Konstantina Kitsou, Anastasia Kotanidou, Dimitrios Paraskevis, Timokratis Karamitros, Aris Katzourakis, Richard Tedder, Tara Hurst, Spyros Sapounas, Athanassios Kotsinas, Vassilis Gorgoulis, Vana Spoulou, Sotirios Tsiodras, Pagona Lagiou, Gkikas Magiorkinis

**Affiliations:** a Department of Hygiene, Epidemiology and Medical Statistics, Medical School, National and Kapodistrian University of Athens, Athens, Greece; b Immunobiology and Vaccinology Research Laboratory, First Department of Peadiatrics, “Aghia Sophia” Children’s Hospital, School of Medicine, National and Kapodistrian University of Athens, Athens, Greece; c 1st Department of Critical Care & Pulmonary Services, Medical School, National and Kapodistrian University of Athens, Athens, Greece; d Unit of Bioinformatics and Applied Genomics, Department of Microbiology, Hellenic Pasteur Institute, Athens, Greece; e Department of Zoology, University of Oxford, Oxford, United Kingdom; f Imperial College, London, United Kingdom; g Birmingham City University, Birmingham, United Kingdom; h National Public Health Organization, Athens, Greece; i Department of Histology and Embryology, School of Medicine, National Kapodistrian University of Athens, Athens, Greece; j Biomedical Research Foundation, Academy of Athens, Athens, Greece; k Center for New Biotechnologies and Precision Medicine, Medical School, National and Kapodistrian University of Athens, Athens, Greece; l Molecular and Clinical Cancer Sciences, Manchester Cancer Research Centre, Manchester Academic Health Sciences Centre, University of Manchester, Manchester, United Kingdom; m 4th Department of Internal Medicine, Attikon University Hospital, Medical School, National and Kapodistrian University of Athens, Athens, Greece; University of Sussex

**Keywords:** COVID-19, endogenous retroviruses, immune senescence, inflammation

## Abstract

Severe COVID-19 pneumonia has been associated with the development of intense inflammatory responses during the course of infections with SARS-CoV-2. Given that human endogenous retroviruses (HERVs) are known to be activated during and participate in inflammatory processes, we examined whether HERV dysregulation signatures are present in COVID-19 patients. By comparing transcriptomes of bronchoalveolar lavage fluid (BALF) of COVID-19 patients and healthy controls, and peripheral blood monocytes (PBMCs) from patients and controls, we have shown that HERVs are intensely dysregulated in BALF of COVID-19 patients compared to those in BALF of healthy control patients but not in PBMCs. In particular, upregulation in the expression of specific HERV families was detected in BALF samples of COVID-19 patients, with HERV-FRD being the most highly upregulated family among the families analyzed. In addition, we compared the expression of HERVs in human bronchial epithelial cells (HBECs) without and after senescence induction in an oncogene-induced senescence model in order to quantitatively measure changes in the expression of HERVs in bronchial cells during the process of cellular senescence. This apparent difference of HERV dysregulation between PBMCs and BALF warrants further studies in the involvement of HERVs in inflammatory pathogenetic mechanisms as well as exploration of HERVs as potential biomarkers for disease progression. Furthermore, the increase in the expression of HERVs in senescent HBECs in comparison to that in noninduced HBECs provides a potential link for increased COVID-19 severity and mortality in aged populations.

**IMPORTANCE** SARS-CoV-2 emerged in late 2019 in China, causing a global pandemic. Severe COVID-19 is characterized by intensive inflammatory responses, and older age is an important risk factor for unfavorable outcomes. HERVs are remnants of ancient infections whose expression is upregulated in multiple conditions, including cancer and inflammation, and their expression is increased with increasing age. The significance of this work is that we were able to recognize dysregulated expression of endogenous retroviral elements in BALF samples but not in PBMCs of COVID-19 patients. At the same time, we were able to identify upregulated expression of multiple HERV families in senescence-induced HBECs in comparison to that in noninduced HBECs, a fact that could possibly explain the differences in disease severity among age groups. These results indicate that HERV expression might play a pathophysiological role in local inflammatory pathways in lungs afflicted by SARS-CoV-2 and their expression could be a potential therapeutic target.

## INTRODUCTION

Human endogenous retroviruses (HERVs) are the remnants of ancient retroviral infections that infiltrated the germ lines of our deep-in-time ancestors and integrated within their genomes. Through Mendelian inheritance and evolutionary processes spanning millions of years, they now occupy around 8% of the human genome (1). Their evolutionary history based on analyses of molecular sequences suggests that at least 30 independent colonization events generated an equivalent number of closely related retroviral integrations known as families (2).

The vast majority of HERV integrations have accumulated mutations that effectively incapacitated their proliferative functionality; however, these integrants can still be transcribed and encode proteins (3). HERV expression is usually silenced through numerous posttranscriptional mechanisms but can be upregulated in certain diseases and conditions, such as cancer and inflammation (4, 5). The retroviral involvement in inflammatory processes has been shown to occur through overproduction of nucleic acids and proteins that interfere with a variety of innate immune response and inflammatory pathways (6).

Coronavirus infectious disease 2019 (COVID-19) emerged in late 2019 in Wuhan City and from China spread around the globe, generating the most intensive pandemic responses within the last 50 years, leading to significant socioeconomic disruption. The disease is caused by a zoonotic coronavirus, now known as SARS-CoV-2, which in a proportion of individuals will cause severe pneumonia and respiratory and multiorgan failure (7). For severe COVID-19, it has been suggested that a critical component of the pathophysiology is a severe inflammatory response driven by cytokine release (8).

Here, we aimed to explore whether HERV expression is upregulated in patients with COVID-19, as this could be consistent with an involvement of HERVs in inflammatory responses such as the production of interferonogenic nucleic acids and inflammatory proteins. In the initial studies, in which the COVID-19 patients’ RNA-seq data were produced, the authors correlated the increased expression of proinflammatory cytokines and cytokine receptors in bronchoalveolar lavage fluid (BALF) samples of COVID-19 patients compared to that in samples of healthy subjects and related this finding to the cytokine storm and disease severity in these patients (9, 10). In addition, we aimed to determine the differences between the expression of HERVs after oncogene-induced senescence of human bronchial epithelial cells (HBECs) in a nonmalignant senescence model and the expression of HERVs in noninduced HBECs (11, 12). In this way, we aimed to determine the effect of senescence on the expression of HERVs as a potential component of the increased inflammatory burden of senescence in bronchial cells (12). We hypothesized that the expression of HERVs is augmented in the state of senescence, and this could offer a plausible explanation for the increased COVID-19 severity and mortality observed as the patient age increases.

We find that specific HERV families are upregulated in BALF of COVID-19 patients compared to those in BALF of healthy individuals but not in the peripheral blood monocytes (PBMCs). The findings merit further study regarding the potential involvement of HERVs in COVID-19.

## RESULTS

### Expression of HERVs in BALF samples and PBMCs from COVID-19 patients in comparison to those from healthy controls.

**FIG 1:**
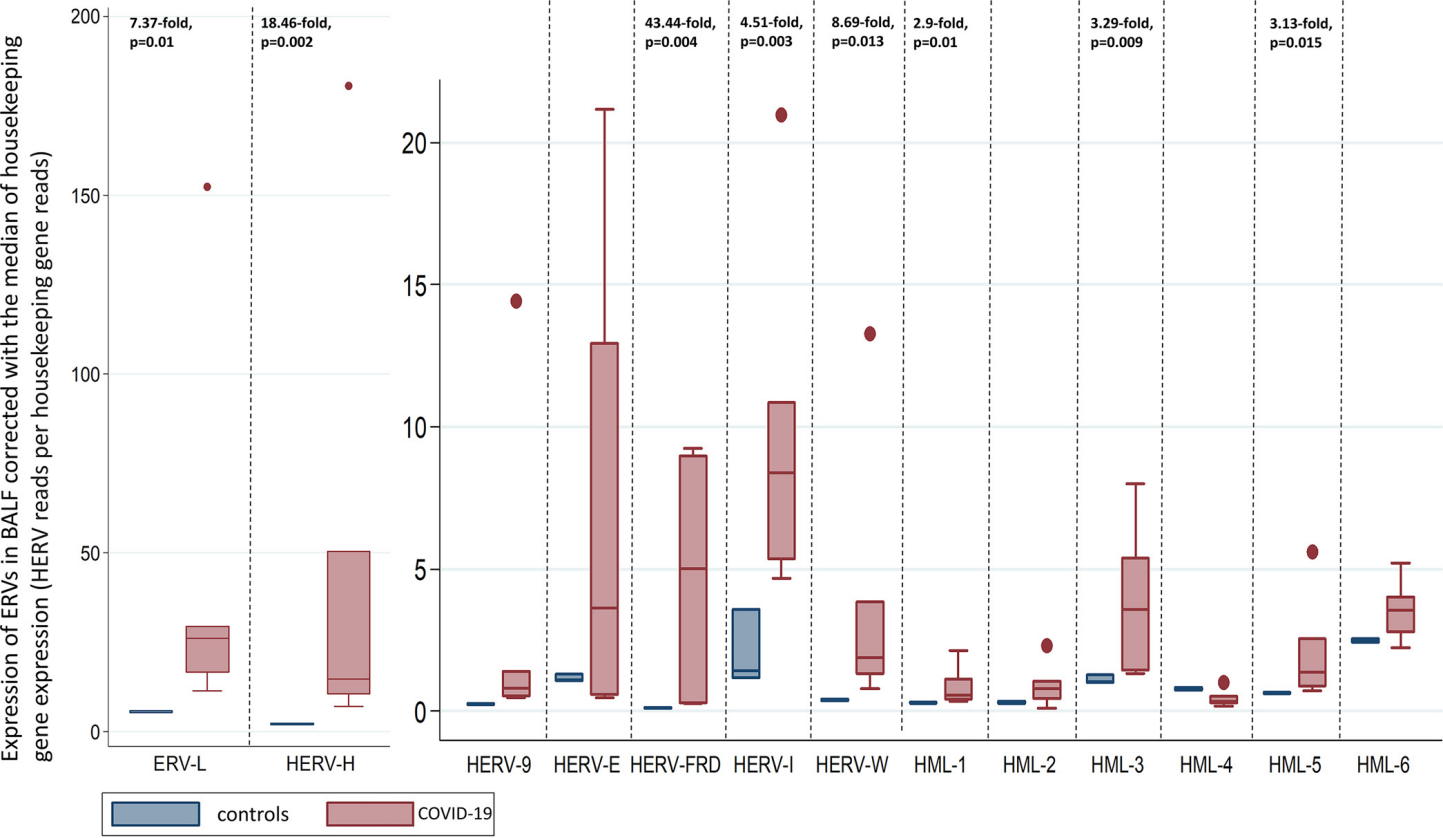
Transcription of HERVs in the BALF of COVID-19 patients and healthy controls corrected with the median of the transcription of four housekeeping genes (SDHA, HPRT1, RBX1, and RRAGA) expressed as HERV reads per housekeeping gene reads. We show statistically significant dysregulation of BALF of COVID-19 patients compared to that of healthy controls as fold change.

We observed an upregulation in the expression of endogenous retroviral elements in the BALF samples of COVID-19 patients in comparison to that in BALF samples of healthy controls. In particular, we observed statistically significant upregulation in the expression of HERV-FRD (43.44-fold change, *P* = 0.004), HERV-H (18.46-fold change, *P* = 0.002), HERV-W (8.69-fold change, *P* = 0.013), ERV-L (7.37-fold change, *P* = 0.01), HERV-I (4.51-fold change, *P* = 0.003), HERV-K (HML-5; 3.13-fold change, *P* = 0.015), HERV-K (HML-3; 3.29-fold change, *P* = 0.009), and HERV-K (HML-1; 2.9-fold change, *P* = 0.01). We observed no statistically significant changes in the BALF of COVID-19 patients compared to BALF samples of healthy controls in the expression of HERV-K (HML-2), HERV-K (HML-4), HERV-K (HML-6), HERV-9, and HERV-E (Fig. 1).

In terms of heterogeneity between the analyzed data sets for the HERVs that were found to be significantly upregulated, the ratios we calculated ranged from 1.42 to 7.19 for HERV-K (HML-1), from 1.19 to 7.16 for HERV-K (HML-3), from 1.10 to 8.54 for HERV-K (HML-5), from 1.91 to 32.23 for HERV-W, from 3.11 to 79.98 for HERV-H, from 2.07 to 27.28 for ERV-L, from 2.27 to 10.17 for HERV-I, and from 2.53 to 85.89 for HERV-FRD.

**FIG 2:**
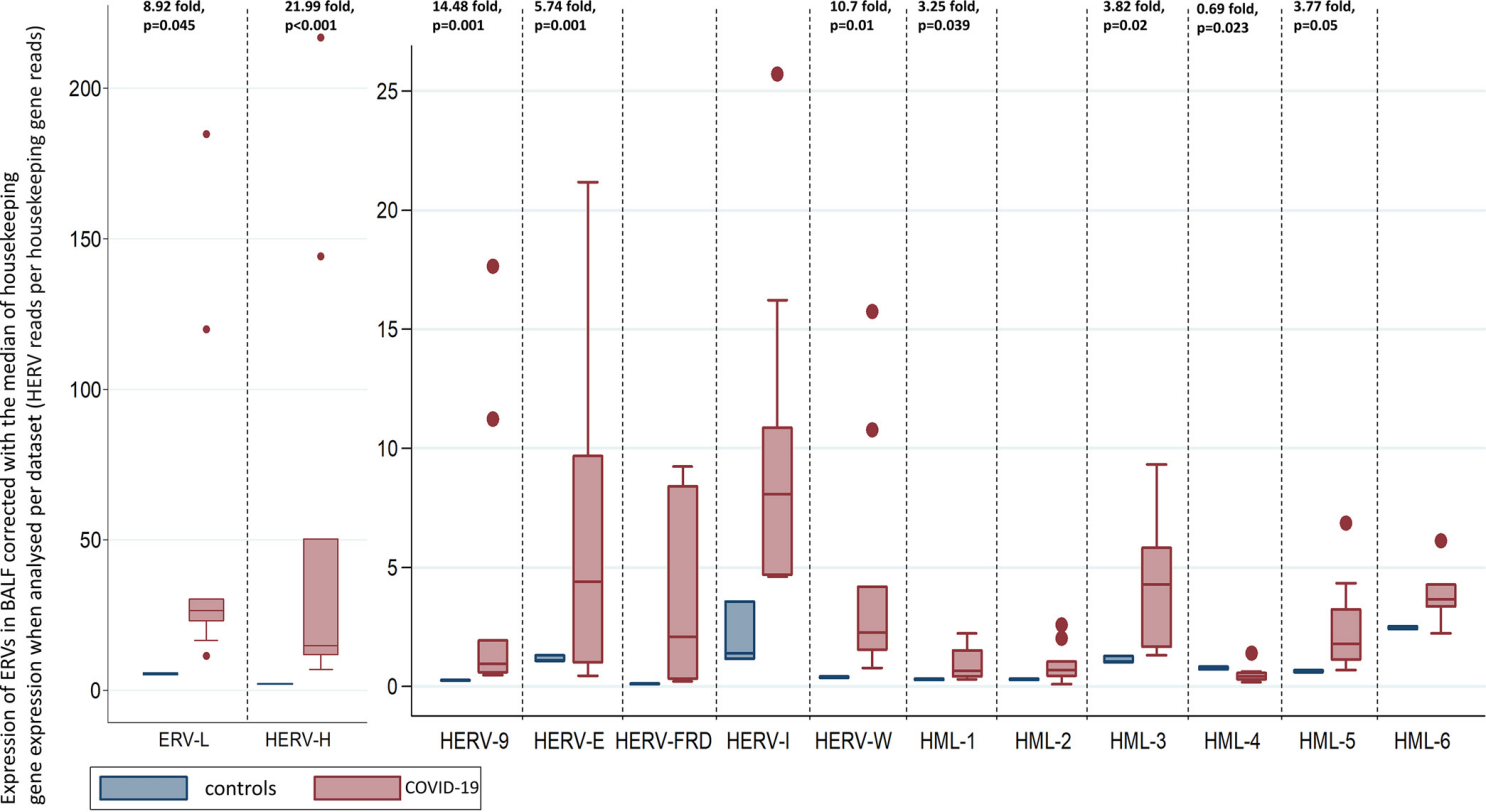
Transcriptions of HERVs in the BALF of COVID-19 patients and healthy controls corrected with the median of the transcription of four housekeeping genes (SDHA, HPRT1, RBX1, and RRAGA) expressed as HERV reads per housekeeping gene reads when each of the biological duplicates available for three out of four COVID-19 patients was analyzed separately.

**FIG 3:**
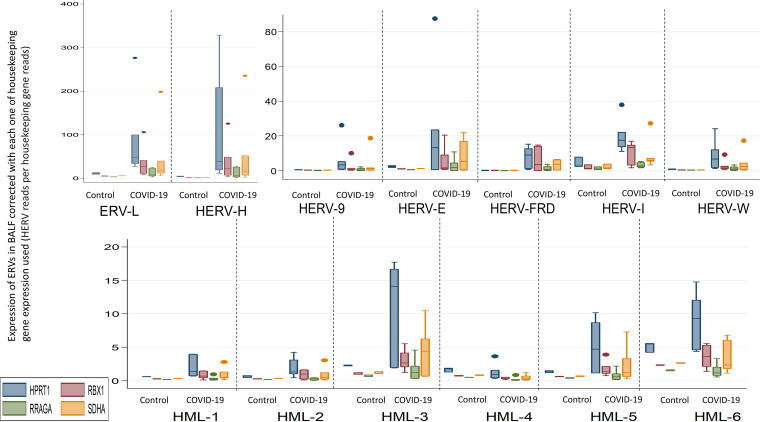
Transcription of HERVs in the BALF of COVID-19 patients and healthy controls corrected with the transcription of each of four housekeeping genes (SDHA, HPRT1, RBX1, and RRAGA) expressed as HERV reads per housekeeping gene reads; each of the corrections is shown separately in box plots with multiple *y* axes simultaneously for the four housekeeping genes.

The results of the sensitivity analyses per data set analyzed and per housekeeping gene are shown in Table 1 (Fig. 2 and 3).

**TABLE 1:** Results of the sensitivity analyses performed on the data of BALF samples of COVID-19 patients in comparison to those of healthy controls per housekeeping gene included as well as per data set included

Sensitivity analysis	HML-1		HML-2		HML-3		HML-4		HML-5		HML-6		HERV-W		HERV-H		HERV-9		ERV-L		HERV-I		HERV-FRD		HERV-E	
	Fold change	*P*	Fold change	*P*	Fold change	*P*	Fold change	*P*	Fold change	*P*	Fold change	*P*	Fold change	*P*	Fold change	*P*	Fold change	*P*	Fold change	*P*	Fold change	*P*	Fold change	*P*	Fold change	*P*
Main analysis	2.9	0.01	2.79	0.244	3.29	0.009	0.56	0.055	3.29	0.015	1.44	0.07	8.69	0.013	18.46	0.002	11.18	0.054	7.37	0.01	4.51	0.003	43.44	0.004	5.52	0.348
SDHA analysis per patient	2.83	0.246	2.68	0.449	3.51	0.126	0.55	0.042	3.36	0.155	1.67	0.071	9.59	0.022	20.37	0.044	12.9	0.17	7.87	0.06	4.07	0.018	27.8	0.002	5.96	0.384
HPRT1 analysis per patient	3.34	0.009	2.79	0.12	4.39	0.035	1.67	0.273	3.87	0.115	1.67	0.071	9.79	0.004	20.7	0.014	11.8	0.014	7.62	0.008	4.67	0.001	33.55	0.001	8.5	0.218
RBX1 analysis per patient	2.87	0.14	2.94	0.157	2.83	0.012	0.5	0.031	2.71	0.028	1.5	0.191	7.37	0.008	16.28	0.014	9.03	0.048	6.74	0.014	5.04	0.036	64.98	0.02	4.79	0.118
RRAGA analysis per patient	1.79	0.515	1.43	0.625	2.24	0.31	0.46	0.015	1.95	0.554	0.95	0.558	4.79	0.09	8.3	0.03	4.51	0.175	3.62	0.044	2.48	0.016	19.35	0.03	3.89	0.616
Analysis per data set	3.25	0.039	3.08	0.177	3.82	0.02	0.63	0.023	3.77	0.05	1.54	0.214	10.7	0.011	21.99	<0.001	14.48	0.001	8.92	0.04	4.7	0.133	35.7	0.384	5.74	0.001

**FIG 4:**
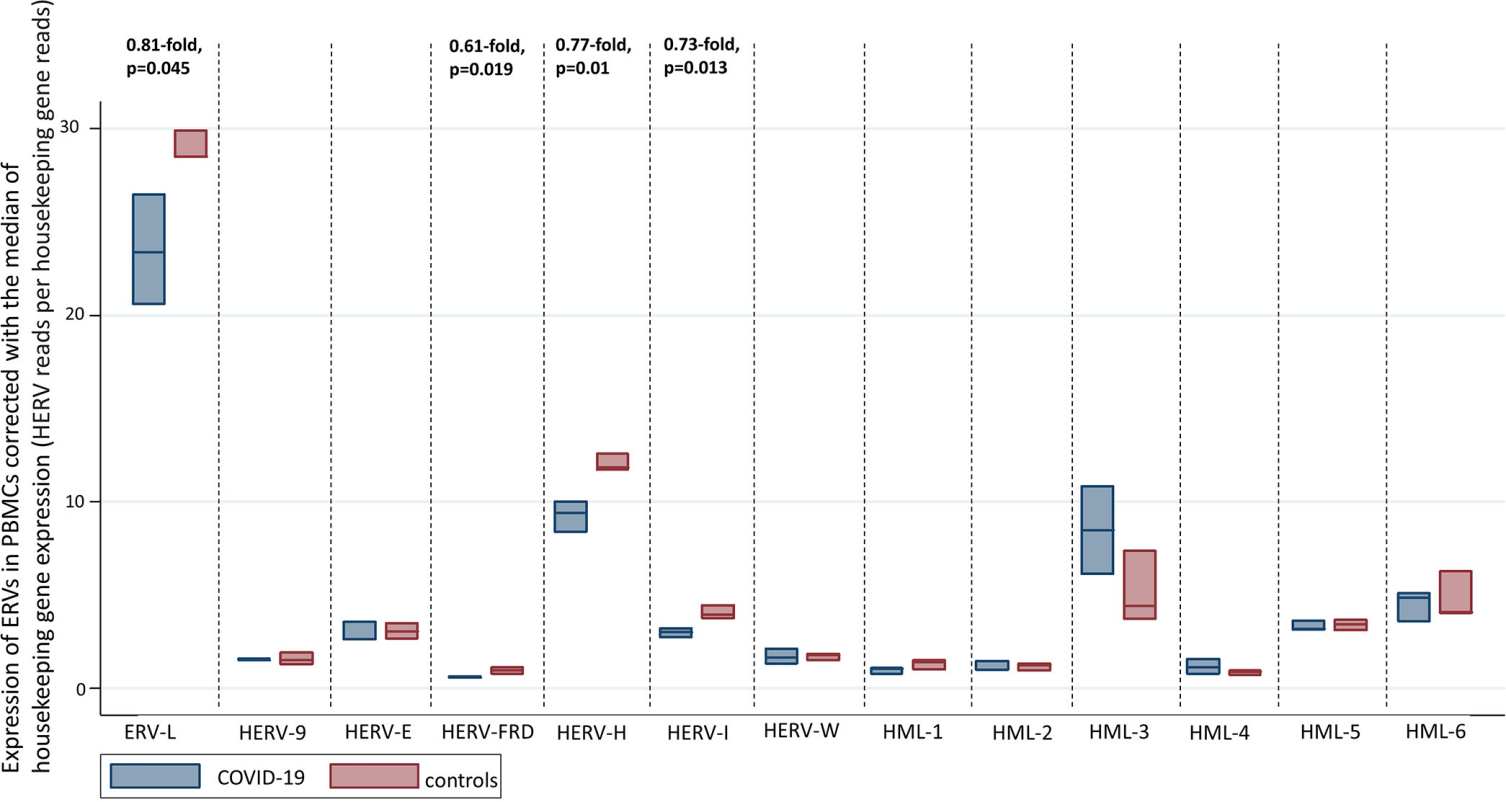
Transcription of HERVs in the PBMCs of COVID-19 patients and healthy controls corrected with the median of the transcription of four housekeeping genes (SDHA, HPRT1, RBX1, and RRAGA) expressed as HERV reads per housekeeping gene reads.

Regarding the expression of endogenous retroviral elements in the PBMCs of COVID-19 patients compared to that in PBMCs from healthy controls, we observed statistically significant downregulation in the expression of ERV-L (0.81-fold change, *P* = 0.045), HERV-FRD (0.61-fold change, *P* = 0.019), HERV-H (0.77-fold change, *P* = 0.01), and HERV-I (0.73-fold change, *P* = 0.013). We observed no statistically significant changes in the expression of HERV-K (HML-1), HERV-K (HML-2), HERV-K (HML-3), HERV-K (HML-4), HERV-K (HML-5), HERV-K (HML-6), HERV-W, HERV-9, and HERV-E (Fig. 4).

**FIG 5:**
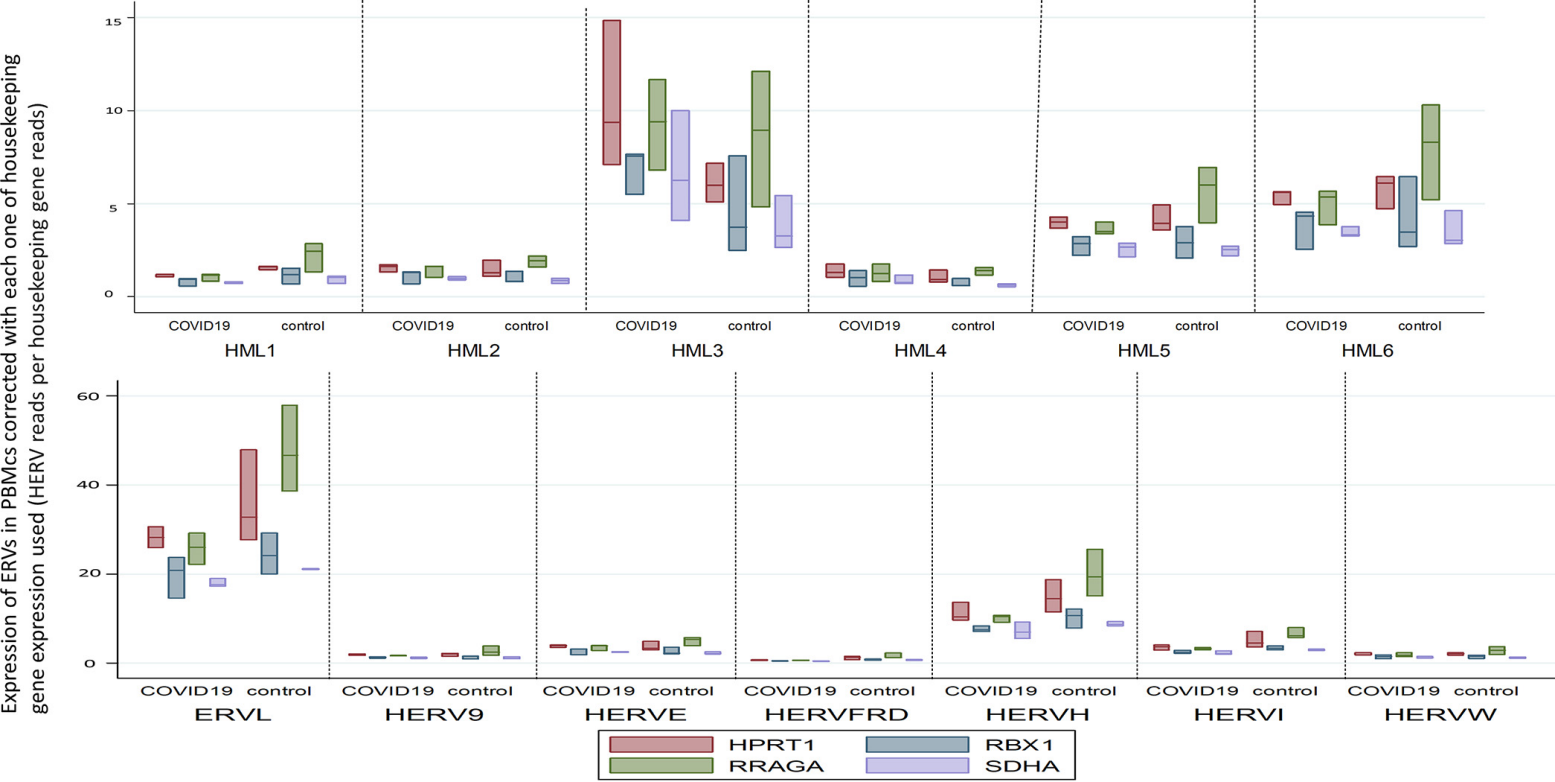
Transcriptions of HERVs in PBMCs of COVID-19 patients and healthy controls corrected with the transcription of each of four housekeeping genes (SDHA, HPRT1, RBX1 and RRAGA) expressed as HERV reads per housekeeping gene reads; each of the corrections is shown separately in box plots with multiple *y* axes simultaneously for the four housekeeping genes.

The results of the sensitivity analyses per housekeeping gene are shown in Table 2 (Fig. 5).

**TABLE 2:** Results of the sensitivity analyses performed on the data of PBMCs samples of COVID-19 patients in comparison to those of healthy controls per housekeeping gene included

Sensitivity analysis	HML-1		HML-2		HML-3		HML-4		HML-5		HML-6		HERV-W		HERV-H		HERV-9		ERV-L		HERV-I		HERV-FRD		HERV-E	
	Fold change	*P*	Fold change	*P*	Fold change	*P*	Fold change	*P*	Fold change	*P*	Fold change	*P*	Fold change	*P*	Fold change	*P*	Fold change	*P*	Fold change	*P*	Fold change	*P*	Fold change	*P*	Fold change	*P*
Main analysis	0.74	0.138	1.12	0.579	1.64	0.124	1.37	0.3	0.98	0.75	0.94	0.795	0.99	0.891	0.77	0.01	0.98	0.915	0.81	0.045	0.73	0.013	0.61	0.019	1.06	0.709
SDHA analysis per patient	0.77	0.153	1.16	0.225	1.79	0.167	1.42	0.147	1.03	0.803	0.98	0.987	1.04	0.825	0.82	0.228	1.03	0.808	0.85	0.029	0.78	0.074	0.64	0.049	1.11	0.292
HPRT1 analysis per patient	0.74	0.007	1.07	0.637	1.72	0.101	1.30	0.301	0.96	0.783	0.94	0.617	0.97	0.821	0.75	0.197	0.97	0.891	0.78	0.26	0.70	0.22	0.59	0.106	1.03	0.776
RBX1 analysis per patient	0.72	0.369	1.11	0.755	1.50	0.216	1.37	0.46	0.95	0.88	0.91	0.859	0.98	0.913	0.75	0.118	0.95	0.922	0.81	0.05	0.73	0.05	0.60	0.032	1.04	0.872
RRAGA analysis per patient	0.48	0.059	0.76	0.177	1.08	0.726	0.93	0.657	0.64	0.077	0.63	0.13	0.65	0.161	0.51	0.014	0.63	0.169	0.54	0.003	0.49	0.003	0.40	0.045	0.71	0.103

### Expression of HERVs in noninduced and induced (senescent) HBECs.

We were able to recognize upregulation in the expression of endogenous retroviral elements in the senescence-induced HBECs in comparison to that in the noninduced HBECs. After the normalization, we detected statistically significant upregulation in the induced HBECs for HERV-K (HML-1; 1.74-fold change, *P* < 0.001), HERV-K (HML-2; 2.11-fold change, *P* < 0.001), HERV-K (HML-3; 1.25-fold change, *P* = 0.002), HERV-K (HML-5; 1.56-fold change, *P* < 0.001), HERV-K (HML-6; 1.2-fold change, *P* < 0.001), HERV-W (1.78-fold change, *P* < 0.001), HERV-9 (1.63-fold change, *P* < 0.001), ERV-L (2.73-fold change, *P* < 0.001), and HERV-E (3.77-fold change, *P* < 0.001). We did not recognize statistically significant differences in the expression of HERV-H and HERV-FRD.

**FIG 6:**
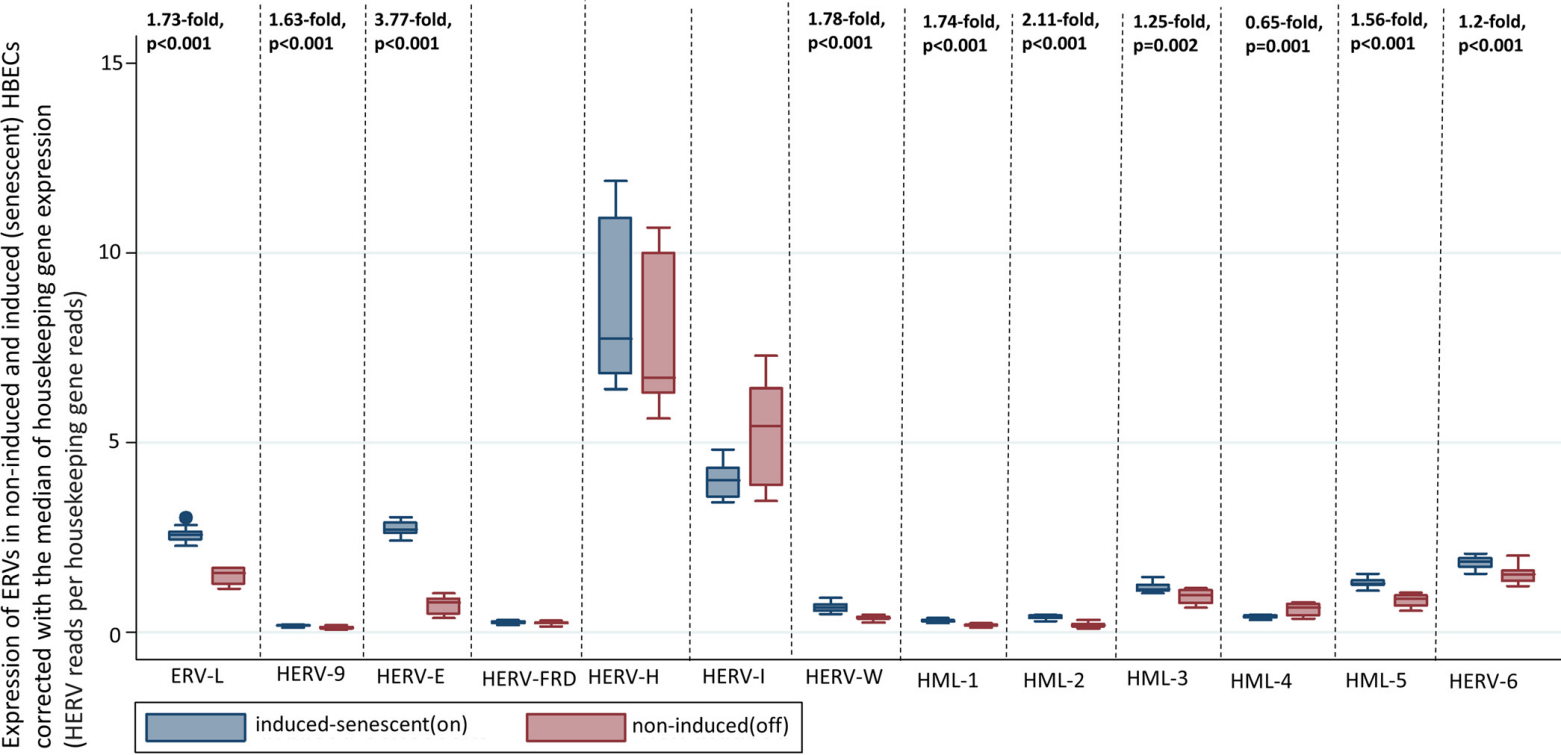
Transcription of HERVs in induced (senescent) HBECs in comparison to that of noninduced HBECs corrected with the median of transcription of four housekeeping genes (SDHA, HPRT1, RBX1, and RRAGA) expressed as HERV reads per housekeeping gene reads, four housekeeping genes (SDHA, HPRT1, RBX1, and RRAGA) expressed as HERV reads per housekeeping gene reads.

Also, we recognized statistically significant downregulation in the expression of HERV-K (HML-4; 0.65-fold change, *P* = 0.001) and in the expression of HERV-I (0.75-fold change, *P* = 0.007) in senescent HBECs in comparison to that in noninduced HBECs (Fig. 6).

**FIG 7:**
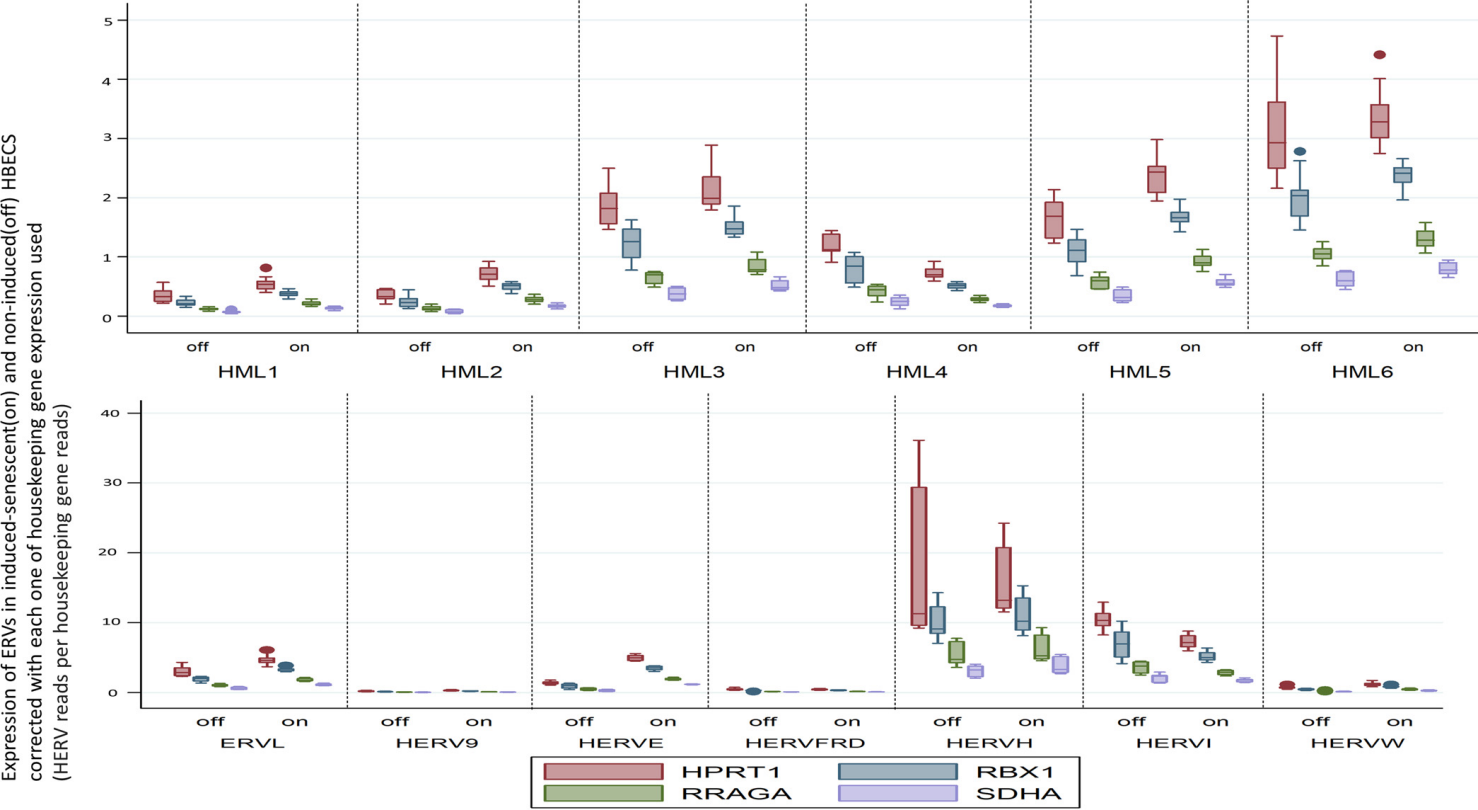
Transcriptions of HERVs in induced (senescent) and noninduced (control) HBECs corrected with the transcription of each of four housekeeping genes (SDHA, HPRT1, RBX1, and RRAGA) expressed as HERV reads per housekeeping gene reads; each of the corrections is shown separately in box plots with multiple *y* axes simultaneously for the four housekeeping genes.

The results of the sensitivity analyses per housekeeping gene are available in Table 3 (Fig. 7).

**TABLE 3:** Results of the sensitivity analyses performed on the data of senescent-induced HBECs in comparison to noninduced HBECs per housekeeping gene included

Sensitivity analysis	HML-1		HML-2		HML-3		HML-4		HML-5		HML-6		HERV-W		HERV-H		HERV-9		ERV-L		HERV-I		HERV-FRD		HERV-E	
	Fold change	*P*	Fold change	*P*	Fold change	*P*	Fold change	*P*	Fold change	*P*	Fold change	*P*	Fold change	*P*	Fold change	*P*	Fold change	*P*	Fold change	*P*	Fold change	*P*	Fold change	*P*	Fold change	*P*
Main analysis	1.74	<0.001	2.11	<0.001	1.25	0.002	0.65	0.001	1.56	<0.001	1.20	<0.001	1.78	<0.001	1.12	0.26	1.63	<0.001	1.73	<0.001	0.75	0.007	1.06	0.415	3.77	<0.001
SDHA analysis	1.91	<0.001	2.29	<0.001	1.36	0.001	0.70	0.011	1.67	<0.001	1.30	0.001	1.90	<0.001	1.23	0.084	1.74	<0.001	1.86	<0.001	0.82	0.079	1.15	0.087	3.98	<0.001
HPRT1 analysis	1.57	<0.001	1.98	<0.001	1.16	0.047	0.61	<0.001	1.43	<0.001	1.08	0.25	1.61	<0.001	0.91	0.914	1.51	0.001	1.57	<0.001	0.70	<0.001	0.95	0.743	3.57	<0.001
RBX1 analysis	1.71	<0.001	2.06	<0.001	1.23	0.009	0.64	0.001	1.53	<0.001	1.18	0.012	1.75	<0.001	1.10	0.29	1.60	<0.001	1.70	<0.001	0.73	0.011	1.04	0.576	3.70	<0.001
RRAGA analysis	1.82	<0.001	2.20	<0.001	1.31	<0.001	0.68	<0.001	1.62	<0.001	1.25	<0.001	1.84	<0.001	1.15	0.229	1.70	<0.001	1.79	<0.001	0.78	0.78	1.10	0.166	3.90	<0.001

## DISCUSSION

SARS-CoV-2 infection is either mild or asymptomatic in the majority of infections (7, 13). Age and underlying comorbidities, like pulmonary and/or cardiovascular disease, are major risk factors for severe COVID-19 (14). However, the underlying causes of the severity of the infection are not fully understood. Here, we have explored whether HERVs are dysregulated in patients with COVID-19, as this could provide evidence in favor of the hypothesis that they could be implicated in the severity of the disease. Based on transcriptomic data, we have found that specific HERV families are upregulated in BALF. Interestingly, BALF samples include a wide variety of cells, such as alveolar macrophages, lymphocytes, neutrophils, eosinophils, and respiratory epithelial cells (15), but this increment cannot be attributed to upregulation in the transcription in PBMCs, as this finding was not reproduced in these cells. Our findings combined with data showing that entry factors for SARS-CoV-2 are coexpressed with innate immunity genes in respiratory cells suggest that HERV upregulation in BALF might indeed be relevant to local rather than systematic immunity responses (16).

While HERVs are not commonly expressed throughout life, they have, on the other hand, been shown to have a dual role with respect to inflammation. They are upregulated by inflammatory pathways (17), but also ERV proteins and nucleic acids may trigger inflammatory responses (18). For example, we have previously shown that IFI-16 has a broad range of single-stranded DNA (ssDNA) targets, and thus if HERVs or other transposable elements are upregulated and reverse transcribed as a result of COVID-19, this could amplify inflammatory responses (19). On the other hand, SARS-CoV-1 and SARS-CoV-2, alike, have been found to delay the production of interferons (20, 21), a mechanism that leads to reduced antiviral responses and hence has been speculated to promote the cytokine storm that has been described in severe COVID-19. In some patients developing severe COVID-19, this immune dysregulation has been linked to the presence of IgG auto-antibodies against type I INFs that preexisted before the infection and to inborn errors in the regulation of type I IFN innate immunity (22, 23). It is reasonable to assume that the intricate mechanisms behind the inflammatory responses to the SARS-CoV-2 infection and its development to severe COVID-19 with cytokine storm in patients prone to severe illness are the result of the aberrant balance in the transcription of proinflammatory, anti-inflammatory, and antiviral immune cytokines, which may be highly influenced by the effect of exogenous and endogenous viral pressure on innate immunity. In addition, the coexistence of external pathogens with endogenous retroelements seems to modulate the endogenous retroviral transcription (24), and thus one can hypothesize that another way in which this cytokine dysregulation occurs in COVID-19 is the presence of SARS-CoV-2 as a trigger for the enhanced transcription of endogenous retroviruses.

In our analyses, HERV-W was one of the most highly upregulated families (up to about 9 times higher). Interestingly, HERV-W *env* has been shown to be associated with proinflammatory transcriptional signatures and to induce proinflammatory responses (25). Whether the upregulation of HERV-W RNA seen in transcriptomes results in production of HERV-W proteins or production of peptides from the retroviral immunosuppressive domain remains to be determined, as the finding of the upregulation at an RNA transcription level might not always result in increased protein translation.

Crucially, HERV expression seems to have an association with age, which is the strongest risk factor for severe COVID-19. Differential expression of HERVs has been described when comparing older and younger adults (26). Upregulation of endogenous retroviruses as a result of aging has been studied in mice, whereas failure of epigenetic mechanisms seems to be the driving force for upregulation of endogenous retroviruses with age (27). Hence, it could be hypothesized that a possible lack of epigenetic regulation of HERVs in older individuals may result in HERV expression overdrive during COVID-19, which in turn results in massive proinflammatory responses (28). This association between aging and increased expression of endogenous retroviral elements is confirmed by our findings. In the framework of this study, an upregulation in the expression of most HERV families included in our analyses is demonstrated in senescence-induced HBECs in comparison to that in noninduced HBECs. These results indicate that the increased expression of endogenous retroviral elements in senescent bronchial epithelial cells possibly induces local chronic inflammation as a hallmark of immunosenescence and enhances the hypothesis of local rather than systematic inflammatory reactions. This hypothesis provides a plausible explanation for the increased COVID-19 severity and mortality as age increases.

Furthermore, COVID-19 exposure has been linked to hyperinflammatory shock in pediatric patients with manifestations that resemble Kawasaki disease (both classic and incomplete); thus, COVID-19 was linked to an unexpected increase of cases of the syndrome (29). Intriguingly, in a case series of Kawasaki pediatric patients linked to the COVID-19 epidemic, HERV levels were detectable in the microbiology results of one of the eight afflicted children described in this case series (29). This finding could potentially indicate a further role of HERVs as an inflammatory trigger upon SARS-CoV-2 infection.

In this analysis, we were able to show a broad spectrum of deregulated expression of specific HERV families. Recognizing the components of this HERV deregulation is a step toward deeper understanding of the pathophysiology behind this dysregulation and its implications in the COVID-19-induced inflammation, as the deregulation observed in this analysis is not uniform among the HERVs included. Crucially, there are HERVs in COVID-19 patients that appear unaffected compared to those in healthy subjects. HERVs as retroviruses have distinct biology and pathophysiology compared to those of transposable or other noncoding elements and produce inflammatory (30) or immunosuppressive proteins (31), form virus-like particles (32), induce the formation of syncytia (31, 33), and protect from exogenous viral infections (32).

The main limitation of our study is the small number of samples; thus, our findings need to be replicated in more patients but also need to be followed up by functional studies. BALF and PBMCs samples are not from the same patients, and thus we have a limitation with respect to direct associations of the expression of endogenous retroviral elements between BALF and PBMCs in COVID-19. First, although different sequencing procedures were used for the production of the data analyzed in our study, we have shown that our method effectively controls for potential mapping differences resulting from different sequencing lengths. It is also striking that HERVs were dysregulated in BALF in every patient studied, while the same pattern could not be observed in PBMCs from any patient studied, suggesting that the association is robust. Finally, we used an oncogene-induced senescence model for lung tissue by which, although one cannot make direct deductions to endogenous retroviral transcription in COVID-19, we aimed to demonstrate the potential causal link between aging and increased COVID-19 severity, as the accumulation of senescent cells in lungs is associated with aging and the senescent cells *per se* are considered to contribute to the aging-associated inflammatory burden (34, 35).

Due to the small number of patients analyzed, we cannot deeply study and analyze potential interperson differences with regard to the induction of transcription of endogenous retroelements in COVID-19. However, we were able to describe heterogeneity among the patients included in this analysis on the size of the fold change of the HERV families’ upregulation, as calculated by the ratio of the individual expression of each of the HERVs to the mean expression in the control samples. One plausible explanation of this heterogeneity in the fold change per data set compared to that of the control mean could be the timing of the sampling of each patient (as there could be different intensity at different time points), as well as the severity of the disease. However, heterogeneity in the expression is expected, and in all the patients we describe, the disruption of the HERV expression has the same directionality. Thus, future studies with numerous patients could potentially elucidate any potential dose-dependent aspect of the upregulation of HERVs in COVID-19.

We find that specific HERV families are upregulated in BALF, but not in PBMCs, in patients with COVID-19 compared to those in healthy individuals. Furthermore, we were able to identify upregulation in the expression of specific HERVs in senescence-induced HBECs in comparison to that of HERVs in noninduced cells, a fact that indicates the potential role of increased endogenous retroviral expression as a mediator of inflammatory reactions in older individuals that are at increased risk due to the disease. The findings merit further study regarding the potential involvement of HERVs in COVID-19. It thus seems feasible that, should HERV expression be etiologically linked to severe COVID-19, this expression could be a therapeutic target that would minimize the likelihood of severe COVID-19 and death. It also remains to be seen if HERV expression could act as a potential marker of disease severity.

## MATERIALS AND METHODS

In order to compare the expression of endogenous retroviral elements in the BALF of COVID-19 patients and healthy individuals and PBMCs of patients and controls, we utilized online available data. For the BALF analysis, we downloaded RNA sequencing (RNA-seq) data from BALF samples of 7 COVID-19 patients (for three of whom two biological duplicates were available for analysis) as well as RNA-seq data from BALF samples of 3 healthy individuals (BALF control data set).

Regarding the RNA-seq data from the BALF samples of COVID-19 patients, we downloaded RNA sequencing data of four COVID-19 patients, CRR119894, CRR119895, CRR119896, CRR119897, HRR057164, HRR057168, and HRR057172, from National Genomics Data Center - Genome Sequence Archive (NGDC-GSA). Additionally, we analyzed single-cell RNA sequencing data of three COVID-19 patients. These data were downloaded from Sequence Read Archive (SRA) with accession numbers SRR13705668, SRR13705698, SRR13705683, SRR13705730, SRR13705665, and SRR13705663. We downloaded RNA-seq data from BALF samples of healthy individuals from Sequence Read Archive (SRA) with accession numbers SRR10571724, SRR10571730, and SRR10571732.

We also downloaded RNA-seq data of PBMCs of 3 COVID-19 patients and 3 healthy donors (PBMC control data set) from National Genomics Data Center - Genome Sequence Archive (NGDC-GSA) with accession numbers CRR119890, CRR119891, CRR119892, CRR119893, CRR125445, and CRR125446.

All data were paired-end, except HRR057164 and HRR057172, which were single-end. The platforms used for the production of the raw data were BGISEQ-500 for CRR119890, CRR119891, CRR119892, and CRR119893 (reads of 100 bases in length), Illumina MiSeq for the production of CRR119894, CRR119895, CRR119896, and CRR119897 (reads of 150 bases in length), Illumina NovaSeq 5000 for the production of CRR125445 and CRR125446 (reads of 150 bases in length), Illumina HiSeq 2500 for the production of HRR057164 (reads of 76 bases length), HRR057168, and HRR057172 (reads of 100 bases in length), and finally Illumina HiSeq 2000 for the production of SRR10571724, SRR10571730, and SRR10571732 (reads of 50 bases in length). Data sets with accession numbers CRR119894 and CRR119895, CRR119896 and CRR119897, and HRR057164 and HRR057168 are biological duplicates and belong to the same patient, respectively. Illumina NovaSeq 6000 was used for the production of SRR13705668, SRR13705698, SRR13705683, SRR13705730, SRR13705665, and SRR13705663, which included paired-end reads with a length of 150 bases. For these data (single-cell RNA-seq), two data sets corresponded to each of the patients included in this study, one of which included CD3^+^ cells (T-cells) and one of which included CD3^−^ cells in the BALF of these patients.

Patients with the samples with accession numbers CRR119891, CRR119892, CRR119893, CRR119894, CRR119895, CRR119896, and CRR119897 are described as severe cases in the initial work (9). The patient whose sample has the accession numbers HRR057164 and HRR057168 is a deceased male patient with an intensive care unit (ICU) admission history due to COVID-19, and the patient whose sample is HRR057172 is a female patient with COVID-19 that did not need ICU admission (10). The patients corresponding to the accession numbers SRR13705668, SRR13705698, SRR13705683, SRR13705730, SRR13705665, and SRR13705663 were male patients with severe COVID-19 (36).

In order to examine the effect of senescence on the expression of ERVs as a potential causal link between aging and increased COVID-19 severity, we conducted an analysis on the expression of HERV families in data from a nonmalignant epithelial oncogene-induced senescence model of production of senescent HBECs as was described by Komseli et al. (11). In a nutshell, in order to study the precancerous and cancerous phases of tumorigenesis in epithelial cells, the researchers developed a platform in which they studied the stages from noninduced human bronchial cells to senescent (induced) and then to “escaped” (cancerous) cells. Noninduced and induced (senescent) HBECs’ online available RNA-seq data were retrieved from NCBI-SRA with accession numbers SRR6261633, SRR6261634, SRR6261635, SRR6261636, SRR6261637, SRR6261638, SRR6261639, SRR6261640, SRR6261641, SRR6261642, SRR6261643, SRR6261644, SRR6261645, SRR6261646, SRR6261647, SRR6261648, SRR6261649, SRR6261650, SRR6261651, SRR6261652, SRR6261653, SRR6261654, SRR6261655, and SRR6261656. The layout of these data sets was paired-end reads with a length of 38 bases, produced in Illumina NextSeq500.

### Bioinformatics analysis.

We used Bowtie2 with default settings for single-end and paired-end data accordingly for the alignment of these data to hg19 human genome assembly (37). We used Samtools view command with the -q option in order for the mapping quality of the reads included in the alignments to be over 20 (38). We used Samtools sort and index commands with default settings. We used integrative genomics viewer (IGV) for the visualization of the mapping alignments (39). We used FastQC for the reads included in this study so that the included data in this analysis have acceptable sequence duplication levels (https://www.bioinformatics.babraham.ac.uk/projects/fastqc/).

For the identification of the expression of endogenous retroviral elements, we used the coordinates of HERV-K (HML-2), HERV-H, HERV-W, HERV-L, HERV-E, HERV-I, HERV-9, HERV-FRD, HERV-K (HML-1), HERV-K (HML-3), HERV-K (HML-4), HERV-K (HML-5), and HERV-K (HML-6) elements, which are described in the existing literature (40–43). HERV-K (HML-2), HERV-H, and HERV-W elements’ coordinates were set in reference genome hg19. Regarding the rest of the endogenous retroviral elements, their coordinates were referring to hg38 assembly, and thus we used the UCSC Batch Coordinate Conversion (liftOver) online tool for the conversion to hg19 coordinates (44).

We used the Bedtools multicov command for the quantification of endogenous retroviral expression in each of the data sets used (45). We utilized the -f option to ensure that at least 80% of the read length was overlapping the HERVs elements’ coordinates. For this reason, based on the coordinates we used and the length of endogenous retroviral elements, we considered a length of 9,000 bp for HML-3, HML-5, and HML-6, 10,000 bp for HML-1, HML-2, HML-4, HERV-W, HERV-L, HERV-E, HERV-I, and HERV-FRD, 12,000 bp for HERV-9, and 17,000 bp for HERV-H for the calculation of -f for each virus in order to increase the sensitivity of the detection. In fact, we created histograms for the distributions for the lengths of the retroelements included in this study and chose to use the lengths that were closer to the longest elements of each virus in order to ensure that reads aligned in longer viral elements would be detected and that we minimized the possibility of losing aligned reads.

Taking into account the different read lengths and different depths of sequencing among the data sets we used in this analysis, we utilized the expression of widely used housekeeping genes for the normalization of our raw reads in each of the data sets. Transcription of HERVs was normalized by means of four housekeeping genes, succinate dehydrogenase (SDHA), hypoxanthine phosphoribosyl transferase 1 (HPRT1), RING-box protein 1 (RBX1), and Ras related GTP binding A (RRAGA). We used the Bedtools multicov command for the quantification of the expression of these genes with the -f option for the detection of reads that overlap the genes’ coordinates by at least 80%, the same way as with the endogenous retroviral elements.

Finally, we calculated the sum of the raw read counts from each ERV in each data set and we normalized the sum of the HERV raw reads by dividing this number by the expression (in raw reads) of each of the housekeeping genes in the respective data set. For the three out of four COVID-19 patients for whom two biological duplicates were available for analysis, the average of the expression, corrected with each of the housekeeping genes used, was calculated and was considered the patients’ expression of the elements tested. Finally, in order to combine the information of the four housekeeping genes used for the needs of this analysis, we calculated and used the median of the expression (normalized with each of the housekeeping genes − normalized expression value) for the main analyses in this work.

For the three single-cell RNA-seq data of the BALF samples for each patient, we added the sums of the raw read counts in CD3^+^ cells and CD3^−^ cells, as well as the raw read counts for the housekeeping genes, in order to obtain a more complete picture of the expression in the BALF sample. We then calculated the normalized expression values as described above.

We calculated the fold change in the expression of the endogenous retroviral elements in COVID-19 BALF samples and PBMCs through the ratio of the mean normalized expression value of ERVs in COVID-19 patients to the mean normalized expression value of ERVs in healthy individuals. Respectively, we calculated the fold change for the expression between noninduced and induced HBECs as the ratio of the mean normalized expression value of ERVs in induced (senescent) HBECs to the mean normalized expression value of ERVs in noninduced HBECs. As a measure of the heterogeneity of expression between the data sets that were included in this analysis, we then calculated the ratio of the expression of each of the HERVs examined in the analyzed data sets to the average expression of the respective HERV in the control data sets.

Also, we have conducted sensitivity analyses with regard to the comparisons per housekeeping gene correction (separately for each one of the housekeeping genes used) for the analyses in BALF, PBMCs, and HBEC data sets. Furthermore, per data sets comparisons with regard to the BALF data sets were also performed. The results for these analyses are included in Tables 1 to 3.

### Statistical analysis.

We used IBM Corp., released 2015, IBM SPSS Statistics for Windows, Version 23.0, Armonk, NY, IBM Corp. for the statistical analysis. The normalized expression value of each ERV was log-transformed (ln[normalized expression value]) in order for us to perform independent sample *t* test, to identify statistically significant differences between the BALF samples and PBMCs of COVID-19 patients and healthy controls, and to identify statistically significant differences in the expression of the studied HERVs between HBECs without and with oncogene-induced senescence. StataCorp. 2009 Stata Statistical Software: Release 11, College Station, TX, StataCorp LP was used for the creation of the figures presented in this work.

### Data availability.

The data sets of the PBMCs from COVID-19 patients and healthy volunteers, as well as the data of BALF from COVID-19 patients analyzed during the current study, are available in National Genomics Data Center-Genome Sequence Archive (NGDC-GSA), in BIG Data Center (https://bigd.big.ac.cn/), Beijing Institute of Genomics (BIG), Chinese Academy of Sciences with accession numbers CRR119890, CRR119891, CRR119892, CRR119893, CRR119894, CRR119895, CRR119896, CRR119897, CRR125445, and CRR125446 (https://bigd.big.ac.cn/gsa/browse/CRA002390) (9).

Data sets of COVID-19 patients’ BALF samples used in this study with accession numbers HRR057164, HRR057168, and HRR057172 are available in National Genomics Data Center-Genome Sequence Archive (NGDC-GSA), in BIG Data Center (https://bigd.big.ac.cn/), Beijing Institute of Genomics (BIG), Chinese Academy of Sciences (https://bigd.big.ac.cn/bioproject/browse/PRJCA002273).

The single-cell RNA sequencing data sets of BALF from patients with severe COVID-19 analyzed as usual RNA sequencing data in the present work are available in NCBI-Sequence Read Archive (SRA) under accession numbers SRR13705668, SRR13705698, SRR13705683, SRR13705730, SRR13705665, and SRR13705663.

The data sets of BALF from healthy controls analyzed in the present work are available in NCBI-Sequence Read Archive (SRA) under accession numbers SRR10571724, SRR10571730, and SRR10571732.

The data sets of the induced and noninduced HBECs analyzed in the present work are available in NCBI-Sequence Read Archive (SRA) under accession numbers SRR6261633, SRR6261634, SRR6261635, SRR6261636, SRR6261637, SRR6261638, SRR6261639, SRR6261640, SRR6261641, SRR6261642, SRR6261643, SRR6261644, SRR6261645, SRR6261646, SRR6261647, SRR6261648, SRR6261649, SRR6261650, SRR6261651, SRR6261652, SRR6261653, SRR6261654, SRR6261655, and SRR6261656.
