# Supplementary material for: Upregulation of Human Endogenous Retroviruses in Bronchoalveolar Lavage Fluid of COVID-19 Patients
Source: Microbiol Spectr. 2021 Oct 6;9(2):e01260-21. doi: 10.1128/Spectrum.01260-21 (PMC8510252; doi:10.1128/Spectrum.01260-21)
Supplement: Supplemental file 1 — Supplemental material. Download SPECTRUM01260-21_Supp_1_seq9.pdf, PDF file, 0.1 MB [file spectrum01260-21_supp_1_seq9.pdf]

Supplemental Material

To test whether the comparison of the expression of endogenous retroviruses is reliable, when it is taking place between datasets generated in different RNAseq platforms and with different read lengths, as we have performed in this work, we used the raw reads extracted in our analysis from one of the datasets used in this work, namely SRR10571730 (one of the BALF controls), to create simulated .fq files imitating the platforms used for the data included in our analysis. This way we demonstrate, that

A) use of family-wide expression rather than integration-specific expression, and
B) normalisations using protein-coding genes

controls for potential sequencing differences among datasets and provides reliable results, even with the use with reads of different read lengths.

First, we simulated the raw reads corresponding to the housekeeping genes used for the normalisations, and to the sequences of HERV-9 (1). Then for each of the simulated data, we proceeded to the creation of new simulated data, where the expression of HERV-9 was increased by three times. We used BBMap tool *randomreads.sh* command for the creation of the fq files for this simulation (2).

In particular, we created 10 separate simulated fastq files. Five of these included the expressions (in raw reads) of HERV-9 and housekeeping genes, observed in SRR10571730, each of which included the following: 76nt long single-end reads, 100nt long single-end reads, 50nt long paired-end reads, 100nt long paired-end reads and 150nt long paired-end reads. The other five fastq files, had the same characterestics (layout and read length) as the former, but each of those showed a 3-fold increase in the initial expression (in raw reads) of HERV-9 -while maintaining the same baseline expression (in raw reads) for the housekeeping genes, we used in our analysis.

We used Bowtie2 command with default settings (3) to map simulated reads in the human genome. We used samtools (4) for processing and then bedtools multicov command (5) to extract the read counts corresponding to each of the housekeeping genes and the HERV-9 loci.

After retrieving the sum of the reads aligned to HERV-9 loci in each of the ten simulated data, all of which had the same baseline expression (in raw reads) of housekeeping genes, we normalized the read counts corresponding to HERV-9 by dividing them to the median of the expression of the housekeeping genes.

The table below shows the normalised expressions per type of reads before and after a three-fold increase in HERV-9 expression:

**HERV9**	**SRR10571730 (control)**	**SRR10571730 (x3)**	**fold increase observed (same parameters)**
**50-paired**	0.278456958	0.83410401	2.995450409
**100-paired**	0.274934301	0.825178326	3.001365498
**150-paired**	0.27578855	0.824481094	2.9895407
**76-single**	0.282028125	0.845568314	2.998170174
**100-single**	0.278763305	0.838063862	3.006363636

This table shows the results after performing the comparisons like they were performed in our main analysis:

**Samples where performed**	**Comparisons made in this work**	**fold increase observed (3x/control)**
BALF	100-p vs 50-p	2.96339632
BALF	150-p vs 50-p	2.96089241
BALF	76-s vs 50-p	3.03662124
BALF	100-s vs 50-p	3.00967111
PBMC	100-p vs 150-p	2.99206884

Thus, the comparison after the three-fold increase in the expression of HERV-9 elements demonstrates no different results regardless of the sequencing technology used for the read generation in each case.

Bias in locus-specific analysis

Although integration-specific expression could reveal important biological features, however we argue that a family-wide analysis for a highly repetitive element, like a HERV family is a technically more appropriate approach for detecting its expression in the human genome. Such an analysis would retrieve a more complete profile of its expression, as the repetitive nature of these elements and the similarities between different integrations could impede the most valid detection of expression across the human genome.

To demonstrate our argument, we randomly selected six HERV-K (HML-2) loci (6). In particular,

chr2	27682845	27683813
chr6	42861409	42871367
chr8	37050885	37051853
chr10	27182399	27183380
chr12	58721242	58730698
chr19	53531160	53532133.

We considered that given the repetitive sequences, these coordinates include, the reads produced in an Illumina platform (in this case of our simulation Illumina HiSeq 2500, single-end lay-out, reads with a length of 150 nt) and correspond to each genomic region – assigned during our simulation, would not directly be matched to their assigned source-sequence, but would rather be “scattered” along the multiple HERV-K (HML-2) integrations sites, as the various integrations sites of an endogenous retrovirus, a highly repetitive element would demonstrate a high similarity to one another.

We used these coordinates to extract the sequences in the Homo sapiens (human) genome assembly GRCh37 (hg19), using Bedtools getfasta command (5). Then we created artificial fastq files, simulating the profiles of Illumina HiSeq 2500 single-end reads, with a length of 150 nt in each file with each of the above sequences having 700, 600, 500, 400, 300 and 200 reads in each corresponding fastq file respectively (7), and hence knowing the number of read counts corresponding as coverage to each HML-2 integration site included in the simulation. After merging these simulated fastq files, for which the read counts were known for each site, we proceeded to mapping in order to detect the alignment of our simulated reads in the human genome, using Bowtie2 with default settings (3). The Bowtie2 output in our file included 2700 reads, as was expected, and an 100% alignment rate to hg19. We, then, retrieved the read counts for each of those HML-2 integrations used for the simulated reads as well as the reads corresponding to all of the HML-2 integration sites in hg19, with the use of the Bedtools multicov command (5).

According to our output 2102 out of 2700 simulated reads aligned to the described HML-2 integrations. More specifically 285 reads aligned within the simulated dataset but on the wrong integration site, while 598 aligned at other integrations. The results from the used coordinates are shown below:

chr2	27682845	27683813	218 aligned out of 700 simulated (31%)
chr6	42861409	42871367	600 aligned out of 600 simulated (100%)
chr8	37050885	37051853	99 aligned out of 500 simulated (19.8%)
chr10	27182399	27183380	468 aligned out of 400 simulated (117%)
chr12	58721242	58730698	417 aligned ouf of 300 simulated (139%)
chr19	53531160	53532133	300 aligned out of 200 simulated (150%)

As it can be seen the bias is strong as the alignment of the reads suggested that the integration on chr2 has lower expression compared to most of the other integrations, while in fact it had the highest. In contrast, the use of the total per family expression as a sum, provides a more complete picture about the

expression of HERV integration sites. Thus, we have concluded that the family-wide analysis is a more suitable approach for analysing short-read sequence datasets to characterize expression of HERVs.

References

1. Bendall ML, De Mulder M, Iñiguez LP, Lecanda-Sánchez A, Pérez-Losada M, Ostrowski MA, Jones RB, Mulder LCF, Reyes-Terán G, Crandall KA, Ormsby CE, Nixon DF. 2019. Telescope: Characterization of the retrotranscriptome by accurate estimation of transposable element expression. PLoS Comput Biol 15.
2. BBMap download | SourceForge.net.
3. Langmead B, Salzberg SL. 2012. Fast gapped-read alignment with Bowtie 2. Nat Methods 9:357–359.
4. Li H, Handsaker B, Wysoker A, Fennell T, Ruan J, Homer N, Marth G, Abecasis G, Durbin R. 2009. The Sequence Alignment/Map format and SAMtools. Bioinformatics 25:2078–2079.
5. Quinlan AR, Hall IM. 2010. BEDTools: A flexible suite of utilities for comparing genomic features. Bioinformatics 26:841–842.
6. Subramanian RP, Wildschutte JH, Russo C, Coffin JM. 2011. Identification, characterization, and comparative genomic distribution of the HERV-K (HML-2) group of human endogenous retroviruses. Retrovirology 8:90.
7. W H, L L, JR M, GT M. 2012. ART: a next-generation sequencing read simulator. Bioinformatics 28:593–594.
